# Reusing larval rearing water and its effect on development and quality of *Anopheles arabiensis* mosquitoes

**DOI:** 10.1186/s12936-016-1227-4

**Published:** 2016-03-16

**Authors:** Wadaka Mamai, Rosemary Susan Lees, Hamidou Maiga, Jeremie R. L. Gilles

**Affiliations:** Insect Pest Control Laboratory, Joint FAO/IAEA Division of Nuclear Techniques in Food and Agriculture, Vienna, Austria

**Keywords:** Sterile insect technique, Mass production, *Anopheles arabiensis*, Larval water

## Abstract

**Background:**

There is growing interest in applying the sterile insect technique (SIT) against mosquitoes. Mass production of mosquitoes for large-scale releases demands a huge amount of water. Yet, many arid and/or seasonally arid countries face the difficulties of acute water shortage, deterioration of water quality and environmental constraints. The re-use of water to rear successive generations of larvae is attractive as a way to reduce water usage and running costs, and help to make this control method viable.

**Methods:**

To determine whether dirty larval water was a suitable rearing medium for *Anopheles arabiensis*, in place of the ‘clean’ dechlorinated water routinely used, a series of three experiments was carried out to evaluate the effect of dirty water or mixed clean and dirty water on several parameters of insect quality. Batches of 100 fresh eggs were distributed in dirty water or added to clean water to test the effect of dirty water on egg hatching, whereas first-instar larvae were used to determine the effect on immature development time, pupation, adult emergence, body size, and longevity. Moreover, to assess the effect of dirty water on larval mortality, pupation rate, adult emergence, and longevity, L4 larvae collected after the tilting or larvae/pupae separation events were returned either to the dirty water or added to clean water.

**Results:**

Results indicated that reusing dirty water or using a 50:50 mix of clean and dirty water did not affect egg hatching. Moreover, no difference was found in time to pupation, larval mortality or sex ratio when first-instar larvae were added to clean water, dirty water, or a 75:25, 50:50 or 25:75 mix of clean and dirty water and reared until emergence. When late-instar larvae were put back into their own rearing water, there was no effect on pupation rate, emergence rate or female longevity, though male longevity was reduced. When reared from first-instar larvae, however, dirty water decreased pupation rate, emergence rate, body size, and adult longevity.

**Conclusions:**

Re-used larval-rearing water has no impact on egg hatching, development time or mortality of the immature stages of *An. arabiensis*. However, dirty water is not suitable for the production of high quality adult mosquitoes. Recycling processes to improve water quality and increase insect quality will be investigated, since it may have important implications for the implementation of the SIT in areas where clean water is a scarce or costly resource.

## Background

Malaria is indisputably one of the most important tropical infectious diseases in humans worldwide, causing approximately 438,000 deaths annually [1]. The enormous loss of lives and days of labour, the costs of treatment of patients, and the negative impact of the disease on development, make malaria a major socio-economic burden [2–4]. No vaccine currently exists, so vector control is a cornerstone in the fight against malaria [5–7]. Methods currently available to control mosquito vectors of malaria rely heavily on the use of insecticides. However, this chemical approach is problematic, with issues such as environmental contamination, effects on non-target organisms and the spread of resistance hampering its effectiveness. Innovative vector control tools are urgently needed, and one such is the sterile insect technique (SIT): male insects are reproductively sterilized with ionizing radiation and released on a regular basis to mate with wild females.

With interest growing in applying the SIT against mosquitoes [8–14], there is a requirement for the development of more efficient and economical methods to produce large numbers of sterile male mosquitoes. Since 2004 the Joint FAO/IAEA Insect Pest Control Laboratory (IPCL) has been working to develop a package of equipment and techniques for the application of the SIT against mosquitoes [15]. The feasibility of the SIT against *Anopheles arabiensis* for malaria control is being tested in Sudan [11, 16] and South Africa [17]. Mass production of mosquitoes demands a huge amount of water, yet these often arid and/or seasonally arid countries face the difficulties of acute water shortage, deterioration or unreliability of water quality and environmental constraints. The United Nations estimates that by 2025 two-thirds of the world population will likely be under water stress [18]. For other water-intensive activities there has been increasing interest in many parts of the world in treating and recycling wastewater, including crop irrigation [19], fish culture [20] and aquatic macrophyte production [21]. The option of re-using water to rear successive generations of mosquito larvae is likewise attractive to reduce water usage and running costs, and help to make the SIT viable.

Immature stages of mosquitoes complete their life cycle in water; the qualities of water required for development vary with mosquito species [22]. Most *Anopheles* mosquitoes species are routinely reared in the laboratory with very clean tap, de-ionized or spring water, but the possibility of re-using *Anopheles* larval-rearing water (hereafter called ‘dirty water’) to rear subsequent generations has not been investigated. Experiments were set out to determine whether dirty water was suitable rearing medium, in place of ‘clean’ dechlorinated water routinely used. Eggs were hatched and larvae reared to pupation in the IPCL-developed larval mass rearing rack [23] until the day pupae were first observed, at which time the racks were tilted to remove the mix of pupae and remaining larvae, keeping the rearing water. The study was set up to test for any effect on several parameters of insect quality: hatch rate, pupation, adult emergence, body size, and adult longevity. A series of three experiments was carried out to investigate: (i) the effect of dirty water on late larval mortality, pupation rate, adult emergence, and longevity when remaining larvae were returned to the rearing water after the first tilting of the rack compared to adding them to fresh, clean water as is current practice; (ii) the effect of dirty water on egg hatching; and, (iii) the effect on immature development and adult quality when larvae completed their whole development, from L1 to pupation, in dirty water, either alone or mixed with some proportion of clean water. In this way the study will allow us to determine to what extent and at what stage in production water could be re-used, without impacting the survival or quality of resulting insects.

## Methods

### Mosquito colonies

Experiments were performed using an *An. arabiensis* Dongola strain originating from the Northern State of Sudan. The colony has been maintained at the IPCL since 2005 under controlled conditions (27 ± 1 °C, 70 ± 10 % relative humidity (RH), 12:12 h light:dark (LD), including 1 h dusk and 1 h dawn. All material, larvae and dirty water, used for this experiment originated from the *An. arabiensis* mass-rearing procedure developed at the IPCL [23–26]. Each tray was filled with 4 L of de-ionized water the day before adding the eggs to allow the water to reach room temperature. Using the egg quantification method developed by Maiga et al. [27], 50 aliquots of 4000 eggs were added to each tray in a plastic ring floating on the surface of the water. Larvae were fed with the IAEA liquid diet (tuna meal: 5 g/L; bovine liver powder: 5 g/L; vitamin mix: 4.6 g/L) following the published protocol [24]. Adult mass rearing cages (200 × 10 × 110 cm) were loaded with around 15,000 pupae, and emerging adult males and females had constant access to 5 % sugar solution using a filter paper (Whatman paper, 2589 A Bogen sheets, 580 × 580 mm). The rearing conditions were 27 ± 1 °C and 70 ± 10 % RH with a 12:12 LD. For egg production, females were given a blood meal using the modified Hemotek membrane feeding system [25] (Discovery workshops, Lancashire, UK). After two blood feedings, gravid females oviposited onto the water surface inside the cages. Eggs were then collected, rinsed and placed on a piece of sterile filter paper, and allowed to air dry for four hours.

### Experiment 1: effect of using dirty water on larval mortality, pupation, adult emergence, and longevity

Larvae were reared as described above, and 24 h after the first pupae were observed the rack was tilted to collect the larvae and pupae for colony maintenance and the used rearing water (‘dirty water’) for this experiment. The ‘dirty’ water was passed through a 50-μm sieve (Retsch^®^ Test Sieve with steel mesh) to remove all eggs, remaining larvae and debris. Pupae were separated from larvae by swirling an Erlenmeyer flask with tap water [28], and larvae (mostly L4) retained for use in experiment 1.

To assess the effect of dirty water on larval mortality, pupation rate, adult emergence, and longevity, the L4 larvae collected after the tilting and larvae/pupae separation events were either returned to the dirty water or added to clean water as is current practice. Approximately 500 late-instar *An. arabiensis* larvae (quantified volumetrically using a modified 50-ml conical centrifuge tube) were placed in plastic laboratory rearing trays (30 × 40 × 7 cm) containing 1 L of either dirty or clean (dechlorinated) water. The clean water was kept for 1 day at room temperature before adding larvae. Three replicates of each treatment were performed. Thirty mL of 1 % IAEA larval liquid diet were added daily to each tray. Total larval mortality was measured as a proportion of initial larvae in each treatment by daily recording all dead larvae in each tray; trays were inspected for dead larvae twice daily to reduce autophagy. Pupae were removed on a daily basis, counted, and placed in 50 ml of the same water in which they had been reared as larvae, and the rate of pupation was calculated based on the total number of pupae counted and removed by the end of the development period. Pupal cups from each replicate were placed in individual cages until all adults emerged to determine emergence rates by subtracting the number of dead pupae. One-hundred male and 100 female adults from each replicate were then transferred to a cage (30 × 30 × 30 cm, BugDorm-1H; MegaView, Taichung, Taiwan) to measure longevity by daily recording and removing dead adults from the cages until all adults had died. A 5 % sugar solution was supplied in a 150-ml plastic bottle with a filter paper.

### Experiment 2: effect of dirty water on egg hatching

This experiment was performed with fresh batches of *An. arabiensis* eggs maintained in mass rearing cages collected from the laboratory colony [29]. One-hundred eggs were distributed to each of 15 transparent cups (diameter 10 cm × height 4.5 cm) containing 100 mL of water lined with filter paper and allowed to hatch; 3 ml of 1 % IAEA diet [24] was added to each cup to stimulate hatching. Five replicates were conducted each with 100 % clean water, 50 % clean water + 50 % dirty water, and 100 % dirty water. First-instar larvae were removed and counted each day after hatching until no new larvae were seen in a cup for three consecutive days, and the total hatch rate was determined.

### Experiment 3: effect of dirty water on immature development and adult longevity

First-instar *An. arabiensis* larvae were obtained by hatching fresh batches of eggs and assigned randomly to different water-type treatments of 500 larvae per laboratory rearing tray (40 × 30 × 7 cm). Each tray was filled with 1 L of one of five water treatments: 100 % clean water, 25 % clean water + 75 % dirty water, 50 % clean water + 50 % dirty water, 75 % clean water + 25 % dirty water, and 100 % dirty water. Each treatment was replicated three times. Larvae were fed 10 mL of a 1 % diet suspension on days 1, 2 and 3, 20 ml on day 4, and then 30 ml daily. Pupae were removed on a daily basis, counted and placed into small bowls containing 50 ml of the same water treatment as they had been reared as larvae. These bowls were put in individual cages (30 × 30 × 30 cm, BugDorm-1H; MegaView, Taichung, Taiwan) until the adults emerged. Larval mortality rate for each treatment was measured by daily removal and counting of all dead larvae from each tray. The rate of pupation for each water type was the total number of pupae obtained at the end of the development period as a proportion of initial L1 number. Dead pupae were counted to calculate the rate of emergence as a proportion of adults emerging from the total number of pupae.

After emergence, 50 males and 50 females from each cage and each water type were transferred to a cage (15 × 15 × 15 cm, Bugdorm.com, Taiwan) for measurement of longevity. The number of males and females emerging was enumerated and used to determine the male/female (M/F) ratios for adults emerging from each treatment. To determine whether larval-rearing water affected adult body size, the right wings of 40–50 females and males per treatment (about 15 wings per replicate) were detached and mounted on glass microscope slides under a cover slip. A photograph of each wing was taken under a dissecting microscope (Leica). Wing length was measured from the tip of the wing (excluding fringe) to the distal end of the alula [30, 31] using analysis^®^ FIVE software. Wing length is considered to be a proxy for mosquito body size [32].

### Statistical analysis

In experiments 1 and 3, pupation rate was calculated as the number of pupae formed, divided by the initial number of larvae, and the emergence rate as the number of adults emerging, divided by the total number of pupae. After angular (arcsinsqrt) transformation of the data expressed as a percentage to stabilize variance and normalize distribution, treatments were compared using either a t test or a one-way ANOVA followed when required by a Tukey’s post hoc test. Visual comparisons of survival curves were estimated using the Kaplan–Meier method. The difference between results from different water-type treatments was compared using the log-rank test. The log-rank test was used to compare the overall longevity trend for the range of treatments explored, and the Gehan-Breslow-Wilcoxon test was used for two-sample comparisons of longevity in each water-type treatment against the longevity in 100 % clean water. The results are given as a test statistic, which was compared with a Chi squared distribution with one degree of freedom to yield a p value. The sex ratio was calculated as the number of emerged adult females divided by the total number of emerged adults (both males and females) for each treatment. For wing length data, the statistical analyses were performed using a one-way ANOVA, followed when required by a Tukey’s post hoc test. All statistical analyses were performed using GraphPad Prism 5.0 software. All treatments were compared against the control: 100 % clean water.

## Results

### Experiment 1: effect of using dirty water on larval mortality, pupation rate, adult emergence, and longevity

Larval mortality did not differ significantly between the dirty and clean water treatments (t test, t = 1.027, df = 2, *P* = 0.4126) with an observed larval mortality of 1.66 ± 0.37 % in clean water and 1.93 ± 0.62 % in dirty water. Neither pupation rate, 98.33 ± 0.37 % in clean water and 98.06 ± 0.62 % in dirty water, nor emergence rate, 93.54 ± 0.82 % and 94.21 ± 1.15 %, respectively, were significantly different between treatments (t test, t = 1.005, df = 2, *P* = 0.4207 and t = 0.418, df = 2, *P* = 0.7165 for pupation and emergence, respectively). All remaining larvae pupated over 4 days after the rack was tilted. The averages time to pupation were 7.109 and 7.131 days for clean and dirty water respectively and there was no difference in time to pupation between treatments (t test, t = 1.248, df = 2, *P* = 0.3006).

**Fig. 1:**
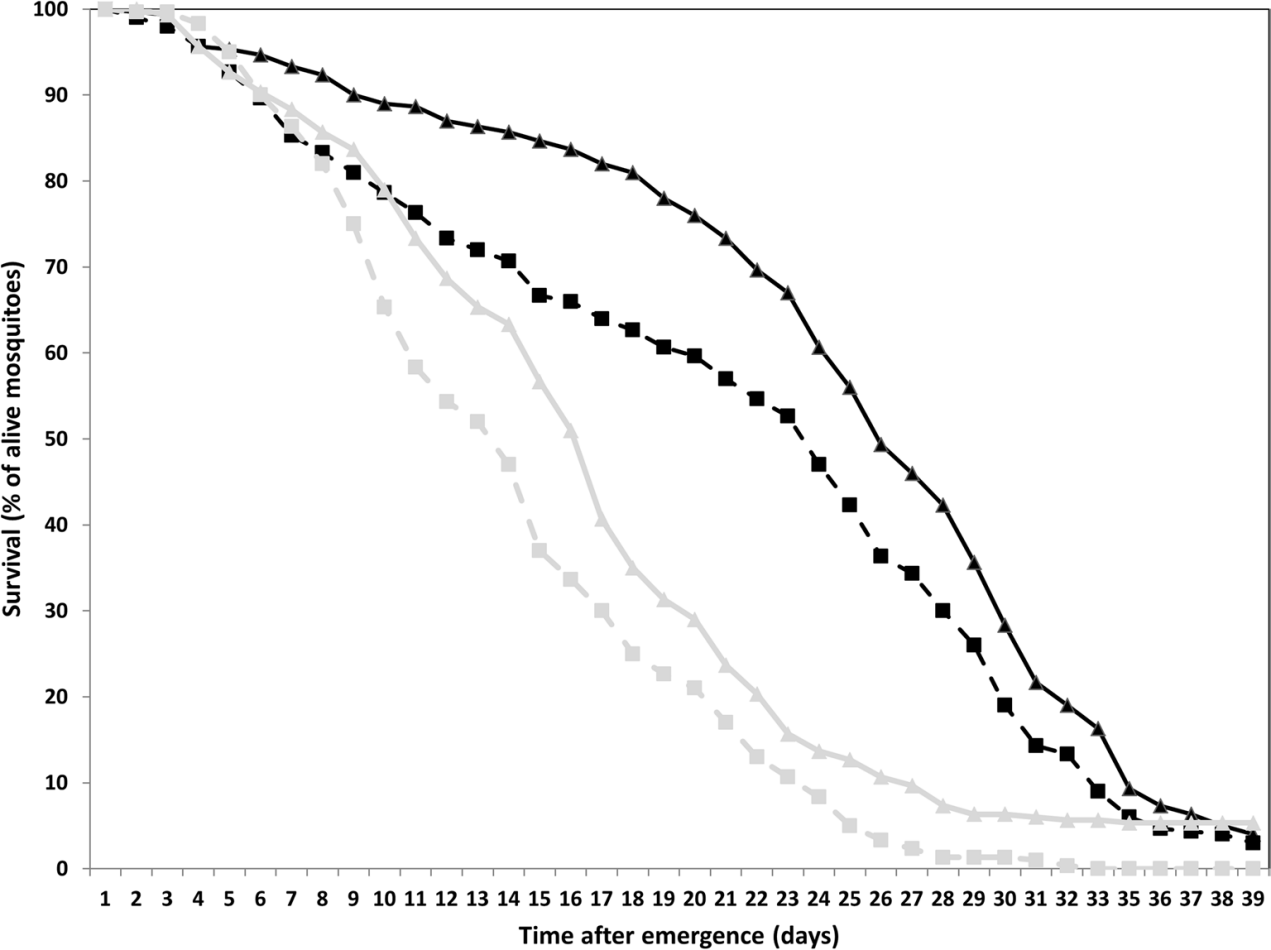
Longevity of males (*black lines*) and females (*grey lines*) of *Anopheles arabiensis* collected from rearing water as late larvae at the start of pupation and reared in dirty (*dashed lines*) or clean water (*solid lines*)

Longevity of males and females was not affected by treatment over the first week post-emergence (graphical observation, Fig. 1) with a low mortality seen in both sexes. After this, mortality was greater in females than in males (Fig. 1). The longevity of males was significantly shorter when maintained in the dirty water (median survival = 23 days) than in clean water (median survival = 25 days) [Log-rank (Mantel-Cox) test, χ^*2*^ = 4.992, df = 1, *P* = 0.0255]. However, dirty water did not have a significant effect on longevity of female adults (median survival = 16 and 13 days in clean and dirty water, respectively, Log-rank (Mantel-Cox) test, χ^*2*^ = 3.257, df = 1, *P* = 0.07). Time to 90 % mortality was 35 and 32 days in males and 26 and 23 days in females in clean and dirty water, respectively. Whether in clean water or in dirty water, males survived longer than females [Log-rank (Mantel-Cox) test χ^*2*^ = 118.5, df = 1, *P* < 0.0001 in clean water and χ^*2*^ = 82.58, df = 1, *P* < 0.0001 in dirty water]. The reduced longevity in females is likely due to absence of blood feeding, indicating that nutrients from blood are essential not only for egg production, but also for survival.

### Experiment 2: effect of dirty water on hatch rate

All eggs in all treatments that hatched did so within 2 days of being submerged. The total hatch rate was not significantly different whether fresh egg batches were added to clean water (62.2 %), dirty water (67.4 %), or a 1:1 mix of clean and dirty water (66.6 %) (ANOVA, F = 0.1935, df = 2, *P* = 0.8266).

### Experiment 3: effect of dirty water on immature development and adult longevity

**Fig. 2:**
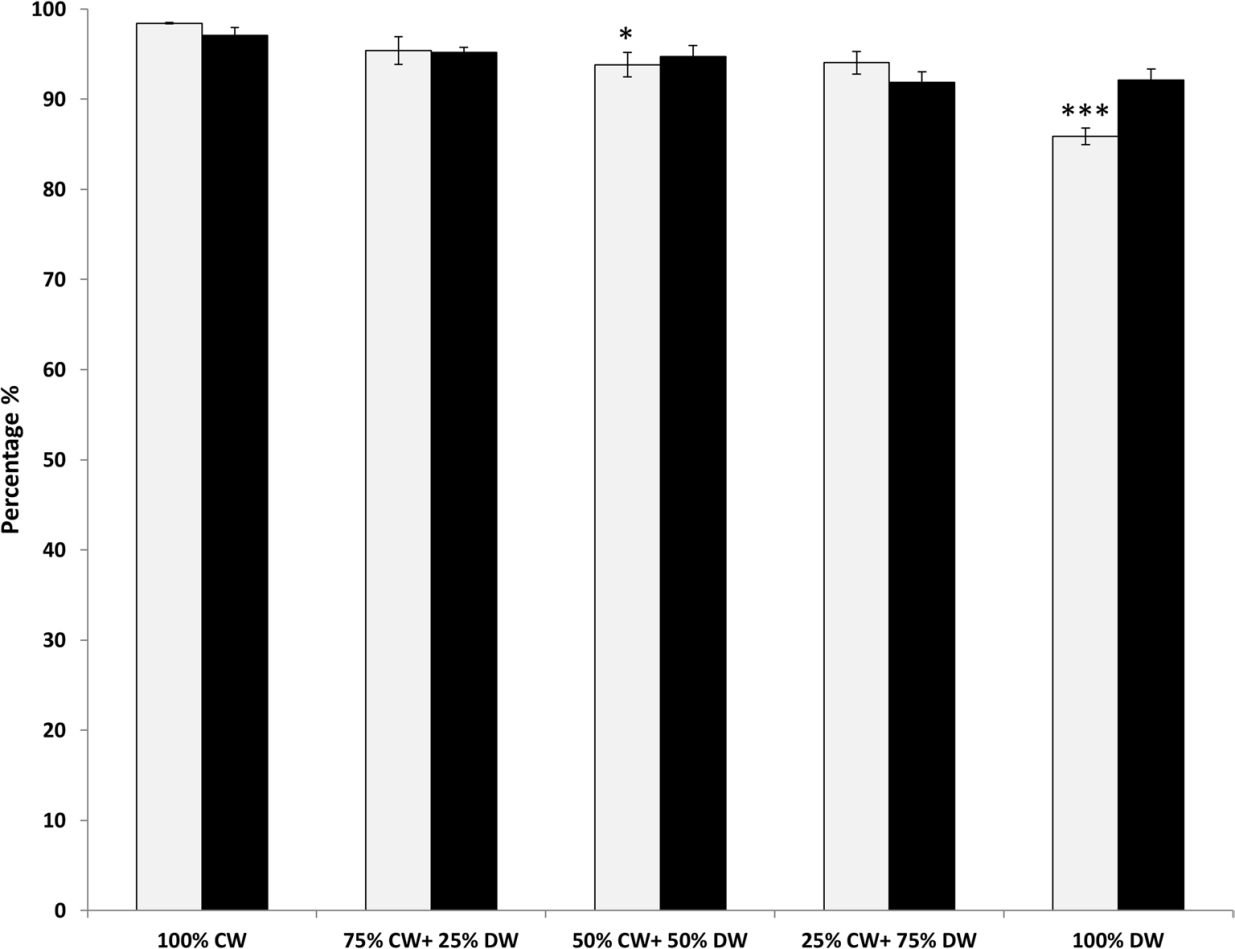
Pupation rate (*grey bars*) and emergence rate (*black bars*) of *Anopheles arabiensis* reared in different larval water treatments. *CW* clean water, *DW* dirty water. *Stars* indicate values statistically different to the 100 % CW control

Pupation rates which ranged from 85.88 ± 0.94 % in 100 % dirty water to 98.43 ± 0.09 % in 100 % clean water were significantly different by treatment (ANOVA, F = 12.97, df = 4, *P* = 0.0006), and significantly lower in 100 % dirty water and the 50:50 mix than in clean water (Tukey’s post hoc test *P* > 0.05). There was a significant difference in emergence rate (ranged from 92.09 ± 1.25 % to 97.06 ± 0.9 %) between treatments (ANOVA, F = 4.5, df = 4, *P* = 0.0245); the rate in 100 % dirty and 25:75 mix treatments were significantly lower than in clean water (Tukey’s post hoc test *P* > 0.05) (Fig. 2). Regardless of water type, pupation started on the seventh day post-hatching, and the majority of larvae (90 %) pupated within 3 days from the first day of pupation. The average time to pupation ranged from 7.69 to 7.97 days, and no difference was found in time to pupation between treatments (ANOVA, F = 0.0086, df = 4, *P* = 0.9998). Male to female sex ratios (ranging between 1:10 to 1:18) did not significantly vary with water type (ANOVA, F = 0.1248, df = 4, *P* > 0.05).

**Fig. 3:**
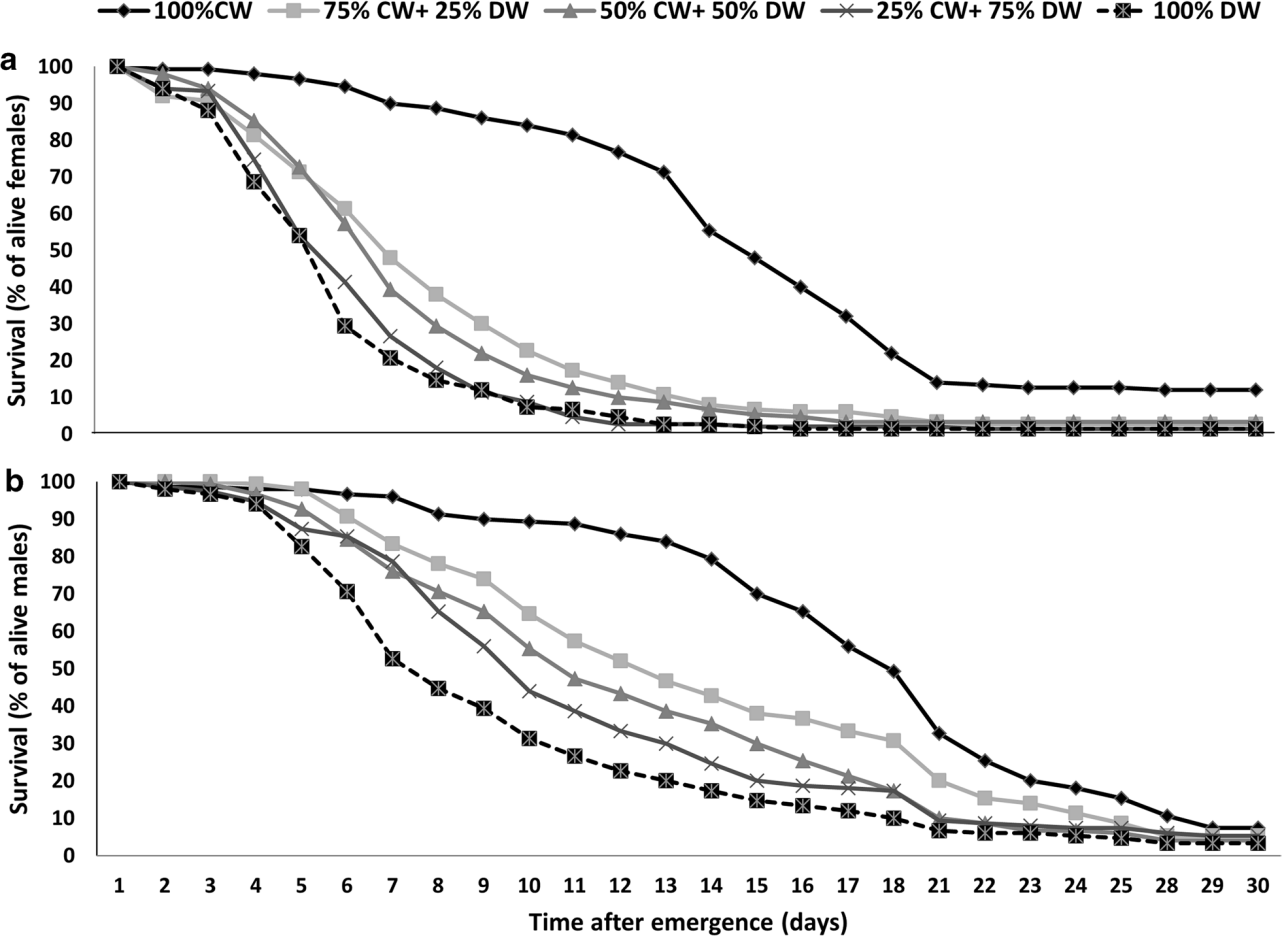
Longevity of male (**a**) and female (**b**) *Anopheles arabiensis* reared from hatching in different larval water treatments. *CW* clean water, *DW* dirty water

Male and female longevity is summarized in Table 1. Rearing larvae in dirty water, even when mixed with clean water, significantly decreased the longevity of adult females (Fig. 3a, log-rank (Mantel-Cox) test, χ^*2*^ = 83.93, df = 4, *P* < 0.0001 and Gehan-Breslow-Wilcoxon tests, *P* < 0.0001) and males (Fig. 3b, log-rank (Mantel-Cox) test, χ^*2*^ = 87.40, df = 4, *P* < 0.0001 and Gehan-Breslow-Wilcoxon tests, *P* < 0.0001).

**Table 1:** Mean (±se) longevity (in days) of *Anopheles arabiensis* males and females reared on different larval water treatments

Sex	100 % CW	75 % CW + 25 % DW	50 % CW + 50 % DW	25 % CW + 75 % DW	100 % DW
Male	17.06 ± 0.50	12.86 ± 0.53	11.21 ± 0.47	10.08 ± 0.46	8.30 ± 043
Female	13.34 ± 0.39	6.82 ± 0.33	6.29 ± 0.25	5.28 ± 0.23	4.94 ± 0.22

*CW* clean water, *DW* dirty water

**Fig. 4:**
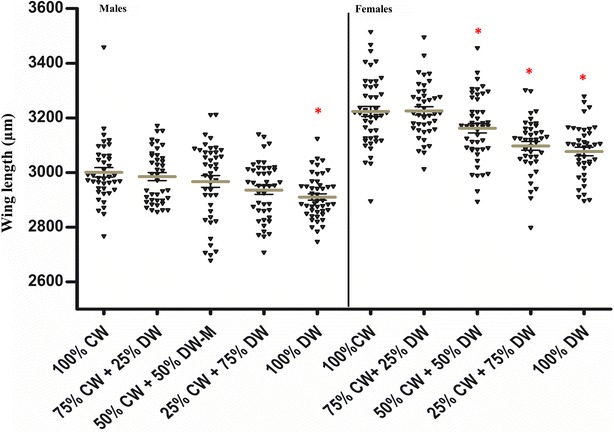
Mean wing-length in male and female *Anopheles arabiensis* reared in different larval water treatments. *CW* clean water, *DW* dirty water. *Stars* signify that the marked value (mean wing length of 45 individuals) was significantly different from the mean measurement of the 100 % CW treatment, by sex

Males from different water treatments showed smaller mean body size (range 2910.46–3000.95 µm) than females (range 3077.24–3227.12 µm). The effect of dirty water during rearing on body size was statistically significant in males (ANOVA, F = 5.062, df = 4, *P* = 0.0006) and females (ANOVA, F = 17.10, df = 4, *P* < 0.0001) (Fig. 4). Using 100 % dirty water resulted in significantly reduced wing length in both males and females (Tukey’s post hoc test, *P* < 0.05), and the 50:50 and 25:75 mixes of clean and dirty water resulted in significantly smaller adult females (Tukey’s post hoc test, *P* < 0.05).

## Discussion

The availability of sufficient water of a reliable quality is an essential requirement for mosquito mass rearing in support of SIT programmes and others relying on large-scale production of mosquitoes. An extensive literature exists describing the characteristics of mosquito larval habitats in nature (e.g., [33–36]). Every *Anopheles* species has its preferred water bodies for oviposition, depending on climate, physical geography and human activities, among other factors. Breeding sites can be natural or man-made, of various sizes, located in running or stagnant waters, shaded or sunny, permanent or temporary. In nature, *An. arabiensis* breeds in small, temporary, clear, and shallow water bodies, with small amounts of organic matter and surface vegetation [37]. It is clear, however, that many species are able to develop and indeed thrive in very dirty water, and so the possibility of re-using water to mass rear successive generations of *An. arabiensis* larvae was tested. By re-using larval-rearing water to reduce the total quantity of clean water required for the rearing process and the amount of waste discharged into the environment, financial savings and a reduced environmental footprint for the rearing facility would be achieved.

In these experiments, neither egg hatching nor larval development time or survival were affected by the use of dirty, re-used, larval-rearing water instead of clean dechlorinated water. Nothing in the re-used water was sufficiently detrimental to development to affect survival or development rate of the immature stages. On the other hand, nothing in dirty water was detrimental for egg hatching and there were no supplemental factors or stimuli in dirty water compared to clean water which contributed to egg hatch. As demonstrated and suggested by many authors [38, 39], many factors other than water temperature (type of water, mechanical agitation, reduced oxygen tension, organic chemicals) affect egg hatching. For example, eggs of *An. diluvialis* hatch poorly in distilled water, but they hatch readily in swamp water and in response to unidentified organic chemicals in an extract from swamp soil [40]. This result is interesting and demonstrates the potential of *An. arabiensis* to exhibit some degree of tolerance to dirty water, consistent with field observations from the *An. gambiae* complex. *Anopheles coluzzii* was found cohabiting with *Culex* species in choke gutters and other organically polluted habitats [36], and it is reported that in some West African cities in the savanna, *An. arabiensis* has developed the ability to breed in larval habitats contaminated by waste waters [41, 42]. So it was not surprising to see successful development of this species in re-used larval-rearing water. However, dirty larval water did have a substantial negative impact on adult size and longevity. Moreover, the low larval mortality rates from L4 onwards indicate that negative effects occur mostly during the early larval instars. Wing length is a reliable correlate of mosquito body size and reflects nutritional status. It has been shown that mosquito size affects egg production [43] and large females are more likely than small ones to successfully oviposit, and lay more eggs [44, 45]. Results revealed that females reared in dirty water have smaller body size, which might further impact their reproductive fitness. Longevity was reduced even in those L4 that were left in their own rearing water for the final days before pupation, relative to the individuals from the same cohort which were transferred to clean water at this stage.

Since the level of adult teneral reserves in mosquitoes is strongly correlated with size and longevity and largely dictated by food assimilation acquired during the immature stages [46], the explanation for the impact of dirty water on adult quality may be reduced nutrition or lower bio-availability in the dirty water relative to the clean water. Hood-Nowotny et al. [47] demonstrated that mosquito size and consequent competitiveness is controlled by the nitrogen content and, maybe more importantly, nutritional bio-availability of that nitrogen from the larval food source. It may be that accumulation of ammonium in larval-rearing water over time depletes available nitrogen until it become limiting, and that moving larvae to fresh water and providing fresh food allows larvae to receive a boost in nutrients which helps adult fitness. A changing bacterial community in the water over time may mean that even though the same diet is being added to the water in each treatment, less actual nutrition is available for larvae due to bacterial competition. Alternatively, the beneficial and nutritional microbes may be depleted by the larvae over time, leading the bacterial community to be dominated by less nutritious organisms. There is evidence that detritus-associated microfauna composition alters over time with larval grazing, though the effect on water column bacteria is less clear [48]. The nutrition available in the dirty water of the current study may have been sufficient to allow development to emergence, but of insufficient quality or missing in specific nutrients required for adults to acquire a good level of teneral reserves.

The natural larval environment of *Anopheles* typically contains algae, bacteria and other micro-organisms, including fungi and protozoa, and the different characteristics of the adults reared in different water treatments may be a result of differences in the micro-fauna present. Many micro-organisms will be exploited by the larvae as a primary source of food [49, 50], and indeed Coon et al. [51] suggest that most, if not all, mosquito species require bacteria to colonize their gut in order to develop to emergence whether in the laboratory or field. Bacteria are mainly acquired during the filter-feeding process where they constitute an essential part of the larval feeding regimen [52, 53]. Some bacteria may improve the digestibility of food [54], and if nutritional or otherwise beneficial organisms may be depleted over time, nutritional availability may also reduce. The diversity of bacteria in mosquito guts reduces during development [55, 56], presumably as beneficial organisms come to dominate, and this may be reflected in their environment. It is also possible that species which may come to dominate over time are toxic or otherwise retard the growth of larvae and impact adult size and performance. Bacterial and other pathogens are certainly a problem in fish and crustacean aquaculture [57, 58], and may also be in mosquito rearing. To better understand whether, and if so how, microbial contribution to larval nutrition affected adult quality it would be interesting to characterize the changing communities over the course of larval development, or at least to compare the composition before and after water is used for rearing, in comparison to water treated similarly but in the absence of mosquito larvae.

Intraspecific larval competition has been shown to reduce adult longevity in *Aedes albopictus* [59] and *An. stephensi* [60], and though most larval competition is through direct resource competition and indirect physical stress, it is possible that interference competition in the form of excreted chemicals exists [61, 62]. Such a retardant factor could have impacted adult survival in larvae reared in dirty water, though Reisen [60] found that *An. stephensi* larvae reared in re-used water developed faster, particularly when the previous larvae had been reared in conditions of low intraspecific competition. He attributed this effect to the presence of more food than in clean water, but also perhaps the presence of autophagostimulants. More likely than some excreted factor, organic pollution from faeces may have been a factor in reducing water quality.

This study has shown that water cannot simply be re-used for production of successive generations of *An. arabiensis* larvae without significant impact on the quality of adult insects. For optimal development larval-rearing water likely needs to meet certain criteria, including optimal pH and dissolved oxygen level, and it is possible that such non-organic factors are responsible for the impact of dirty water. However, it may be possible to treat the water in some way before it is re-used. The method used would depend on what qualities of the used water are detrimental to larval development, and needs to be quick, easily applied to large volumes of water and not add substantial costs to the rearing process. Lessons could perhaps be learned from industrial closed- or recirculating-system aquaculture of other species, where water re-use has been considered for many years [63]. For example, Otte and Rosenthal [64] used a trickling filter, ozonation treatment, and a glucose and methanol denitrification unit to produce water of sufficient quality for high-density fish culture in brackish water. Biological treatment is considered to be the least costly approach, and can manage the main water quality problems of oxygen depletion and accumulation of organic matter, nitrates and carbon dioxide [63]. Although relying on plant or algal denitrification or bacterial decomposition would not be practical in mosquito culture, biofilters have been used to recycle water for aquaculture, to remove excess nitrogen and carbon [65], and may be a practical solution in combination with agitation for aeration. Such filters have been developed for treating potable water in disaster scenarios or for travellers [66], and so should be economical and effective enough to use for this purpose. Physical filters would need to be less than 5 µ in aperture to remove bacteria, which would be very expensive to implement. Alternative methods may include irradiation such as UV [66], ultrafiltration [67], or ultrasonic treatments [68], which could even be driven by sunlight [69], heat treatment, irradiation or autoclaving, if appropriate engineering solutions can be developed to be practical in a programmatic setting. Bacterial communities are likely to vary between rearing trays and between generations, and so mixing water from all trays in the rack before treatment and re-use avoids introducing this as a variable into the mass-rearing process in the next generation. If specific bacteria are found to be advantageous to larval rearing, they could be added to the rearing water as a probiotic to enhance development, and in re-used water to help beneficial organisms to dominate over any detrimental ones.

## Conclusions

*Anopheles arabiensis* can be reared in water previously used for this purpose without any effect on hatch rate, larval development time, or mortality. However, this ‘dirty’ water negatively influenced body size and longevity in emerging adults, so that although production of mosquitoes was not affected by re-using rearing water, the quality of the subsequent adults was affected. The reason for the reduced longevity is not presently known, and may be either due to nutritional status of the water or due to substances in, or lacking from, the water, which are detrimental to development. Further investigation will be carried out to determine the potential causes (e.g., lowered oxygen concentration, bacterial composition or pollution) for the reduced longevity. It may be possible to develop an appropriate treatment for the water to make it suitable for re-use for subsequent generations of larvae. The findings of this study should serve as baseline information for the development of recycling water to meet larval water quality needs for optimal mosquito development and subsequent fitness.

## Abbreviations

SIT: sterile insect technique
WHO: World Health Organization
RH: relative humidity
IPCL: Insect Pest Control Laboratory
FAO: Food and Agricultural Organization
IAEA: International Atomic Energy Agency
LD: light:dark
